# Usefulness of the frequency-volume chart over the International Prostate Symptom Score in patients with benign prostatic hyperplasia in view of global polyuria

**DOI:** 10.1371/journal.pone.0197818

**Published:** 2018-07-11

**Authors:** Sangjun Yoo, Juhyun Park, Sung Yong Cho, Min Chul Cho, Hyeon Jeong, Hwancheol Son

**Affiliations:** 1 Department of Urology, Seoul National University Boramae Medical Center, Seoul, Republic of Korea; 2 Department of Urology, Seoul National University Hospital, Seoul National University College of Medicine, Seoul, Republic of Korea; Cedars-Sinai Medical Center, UNITED STATES

## Abstract

**Purpose:**

We aimed to determine the usefulness of the frequency-volume chart over the International Prostate Symptom Score in patients with benign prostatic hyperplasia. Furthermore, we investigated the clinical characteristics suggesting that patients could benefit from frequency-volume chart assessment in addition to International Prostate Symptom Score assessment.

**Methods:**

A total of 193 patients with benign prostatic hyperplasia were analyzed. The relationship between the information obtained from the frequency-volume chart and the International Prostate Symptom Score was assessed. Because the urine output per kilogram per hour was not associated with any question in the International Prostate Symptom Score questionnaire, patients were divided into 2 groups according to the presence of global polyuria, defined as urine output >40 mL·kg^-1^·h^-1^. Multivariable analysis was performed to determine the predictors of global polyuria, and the results were externally validated using 397 patients with benign prostatic hyperplasia.

**Results:**

Although the other information obtained from the frequency-volume chart correlated with the International Prostate Symptom Score, the urine output was not associated with the International Prostate Symptom Score. Based on these results, patients were dichotomized into the global polyuria group (n = 19, 9.8%) and the non-global polyuria group. Although the patient characteristics did not differ between the 2 groups, the number of voids was higher in patients with global polyuria. Multivariable analysis showed that diabetes mellitus (odds ratio: 3.497, *p* = 0.039) and increased number of voids (odds ratio: 1.320, *p* < 0.001) were significant predictors of global polyuria. On external validation, the area under curve for the model was 0.723.

**Conclusions:**

Global polyuria cannot be suspected using the International Prostate Symptom Score, although it worsens the lower urinary tract symptoms of patients with benign prostatic hyperplasia. Assessment with the frequency-volume chart needs to be considered in diabetic patients with increased number of voids.

## Introduction

Lower urinary tract symptoms (LUTS) due to benign prostatic hyperplasia (BPH) are reportedly associated with reduced quality of life (QoL) [1]. Therefore, subjective symptoms are important factors in assessing the severity of LUTS in male patients, in addition to objective urinary parameters. In this context, several questionnaires have been introduced to evaluate the characteristics and degree of LUTS in patients with BPH [2–4]. Among such evaluation tools, the International Prostate Symptom Score (I-PSS) is considered the most reliable and most frequently assessed index in male patients with LUTS who are suspected to have BPH [5]. However, the I-PSS has several limitations, including misclassification of symptoms [6]. In addition, the I-PSS showed a weak correlation with prostate volume and objective urinary parameters derived from other examinations [7–9].

To adequately assess voiding symptoms, causes of LUTS, and their consequences, a number of evaluations need to be performed based on the patient’s clinical profile [10]. Among these, the frequency-volume chart (FVC), a record of the time and volume of every void measured for a certain period, contains a huge amount of information about voiding symptoms and patterns that cannot be assessed using other examinations. However, in male patients with LUTS, the current clinical guidelines recommend FVC assessment only in patients with a predominant storage symptom and/or nocturia [10, 11]. Consequently, FVC assessment is likely to be selectively performed according to the clinician’s decision considering the patient’s clinical characteristics.

Although some studies have aimed to evaluate the value of the FVC in patients with LUTS [12, 13], the usefulness of routine FVC assessment over I-PSS assessment has not been well evaluated until now, despite the huge amount of information that can be obtained only by using the FVC, as mentioned above. Therefore, we compared the information obtained from the FVC with the score of each question of the I-PSS questionnaire and the sum of I-PSS, in order to assess the usefulness of the FVC over the I-PSS in patients with BPH. In addition, we assessed the characteristics of patients who could potentially benefit from FVC assessment in addition to I-PSS assessment to suggest more relevant selection criteria for FVC in male patients with LUTS who are suspected of having BPH.

## Materials and methods

This study was approved by the Institutional Review Board of Boramae Medical Center. A total of 876 patients who underwent transurethral surgery for BPH between January 2008 and January 2017 were initially included in the study. Among these patients, 214 patients who completed both FVC and I-PSS questionnaire assessments at any time before surgery were selected. After excluding 19 patients with a duration between FVC and I-PSS assessments of >3 months and 2 patients with a period of FVC recording of <72 hours, 193 patients were finally included in the analysis. The median duration between the FVC and I-PSS questionnaire assessments was 0.4 months (interquartile range, 0.2–0.9 months). In addition, for the external validation, the medical records of 397 patients with BPH who visited the outpatient clinic at Boramae Medical Center and underwent FVC assessment were retrospectively reviewed.

Generally, all patients with suspected BPH were assessed using the I-PSS questionnaire at the first visit. The questions in the I-PSS questionnaire were as follows: question (Q)1, incomplete emptying; Q2, frequency; Q3, intermittency; Q4, urgency; Q5, weak stream; Q6, straining; Q7, nocturia; and QoL. FVC assessment was performed if clinically needed according to the patients’ symptoms and clinical characteristics. The FVC was recorded during 72 hours and was interpreted by the clinicians. The following information was obtained from the FVC: 24-hour urine output (UO), nocturnal UO, number of daytime voids, number of nocturnal voids, and maximum voided volume (MVV). The 24-hour UO was defined as the mean daily voided urine volume during a 3-day period, and nocturnal UO was defined as the mean nocturnal voided urine volume including the first morning void during a 3-day period. MVV was defined as the largest single voided volume during a 3-day period. Nocturnal polyuria index (NPi), nocturia index (Ni), and nocturnal bladder capacity index (NBCi) were calculated using the following formulas: NPi = nocturnal UO / 24-hour UO, Ni = nocturnal UO / MVV, and NBCi = number of nocturnal voids—Ni—1.

The patients’ characteristics were expressed using a frequency table for categorical variables and means ± standard deviations for continuous variables. We aimed to assess the information derived from the FVC that could not be inferred from the results of the I-PSS questionnaire. Therefore, 24-hour UO, nocturnal UO, MVV, number of daytime voids, and number of nocturnal voids were compared according to the score of each question in the I-PSS questionnaire using the analysis of variance test. In addition, hourly UO (mL/h) divided by body weight (kg) was compared according to the score of each question in the I-PSS. As hourly UO (mL/h) divided by body weight (kg), in addition to 24-hour UO, was not associated with any question in the I-PSS questionnaire, the patients were divided into 2 groups according to the presence of global polyuria, defined as UO ≥ 40 mL·kg^-1^·h^-1^ [14], and the patients’ characteristics were compared between the 2 groups. Univariate and multivariable logistic regression analyses were performed to determine the predictors of global polyuria. Variables with *p* < 0.2 in the univariate analysis were included in the multivariable analysis, and the backward elimination method was used for the multivariable analysis. The developed prediction model was externally validated, and the area under the curve (AUC) of the receiver operating characteristic (ROC) curve for the prediction model was calculated. All statistical comparisons were performed using IBM SPSS Statistics version 21 (IBM SPSS, Armonk, NY, USA) and R statistical package 3.3.2 (https://www.r-project.org). A value of *p* < 0.05 was considered statistically significant.

## Results

The mean age of the patients was 68.0 ± 8.5 years, and the mean body mass index was 24.0 ± 3.1 kg/m^2^ (Table 1). Hypertension and diabetes mellitus was detected in 78 (40.4%) and 36 (18.7%) patients, respectively. The mean prostate volume measured using trans-rectal ultrasonography was 50.8 ± 27.8 mL. The mean score of each question in the I-PSS questionnaire was as follows: Q1, 2.9 ± 1.8; Q2, 3.0 ± 1.6; Q3, 2.9 ± 1.8; Q4, 2.5 ± 1.8; Q5, 3.6 ± 1.7; Q6, 2.6 ± 1.9; Q7, 2.7 ± 1.4; and QoL, 4.1 ± 1.5. The mean sum of the I-PSS was 20.0 ± 9.1. The number of daytime and nocturnal voids was 8.3 ± 2.9 and 1.7 ± 1.2, respectively. The mean 24-hour UO and nocturnal UO was 1651.0 ± 624.6 mL and 522.9 ± 248.3 mL, respectively. The MVV was 323.7 ± 133.1 mL. The NPi, Ni, and NBCi were 31.9 ± 10.4%, 1.8 ± 2.2, and 0.9 ± 2.1, respectively.

**Table 1:** Patient characteristics according to the presence of global polyuria.

	Total	Without global polyuria	With global polyuria	*p*
Number of patients, n (%)	193 (100.0)	174 (90.2)	19 (9.8)	
Age, years, mean ± SD	68.0 ± 8.5	67.8 ± 8.8	70.3 ± 5.7	0.231
Diabetes, n (%)	36 (18.7)	30 (17.2)	6 (31.6)	0.128
Hypertension, n (%)	78 (40.4)	73 (42.0)	5 (26.3)	0.187
BMI, kg/m^2^, mean ± SD	24.0 ± 3.1	24.2 ± 3.1	22.8 ± 2.8	0.060
PSA, ng/mL, mean ± SD	6.7 ± 16.7	6.8 ± 17.4	6.1 ± 7.3	0.880
Prostate volume, mL, mean ± SD	50.8 ± 27.8	50.4 ± 26.6	54.6 ± 37.7	0.529
Number of voids, n, mean ± SD				
Daytime	8.3 ± 2.9	8.0 ± 2.5	11.2 ± 4.3	<0.001
Nocturnal	1.7 ± 1.2	1.7 ± 1.1	2.5 ± 1.5	0.002
24-hour urine output, cc, mean ± SD	1651.0 ± 624.6	1517.7 ± 474.6	2872.3 ± 500.0	<0.001
Nocturnal urine output, cc, mean ± SD	522.9 ± 248.3	491.1 ± 217.5	813.5 ± 322.6	<0.001
Maximum voided volume, cc, mean ± SD	323.7 ± 133.1	315.4 ± 123.0	399.5 ± 191.9	0.009
Nocturnal polyuria index, %, mean ± SD	31.9 ± 10.4	32.3 ± 10.3	28.4 ± 10.8	0.115
Nocturia index, mean ± SD	1.7 ± 0.8	1.7 ± 0.7	3.7 ± 6.6	<0.001
NBCi, mean ± SD	1.0 ±7.2	1.0 ± 0.7	-0.1 ± 6.4	0.027
The I-PSS questionnaire				
Q1	2.9 ± 1.8	2.9 ± 1.8	3.1 ± 1.8	0.570
Q2	3.0 ± 1.6	2.9 ± 1.6	3.6 ± 1.8	0.078
Q3	2.9 ± 1.8	2.8 ± 1.8	3.7 ± 1.8	0.031
Q4	2.4 ± 1.8	2.4 ± 1.7	3.1 ± 2.0	0.082
Q5	3.6 ± 1.7	3.5 ± 1.7	4.2 ± 1.5	0.139
Q6	2.6 ± 1.9	2.6 ± 1.9	2.8 ± 2.1	0.560
Q7	2.7 ± 1.4	2.6 ± 1.3	3.3 ± 1.5	0.054
Sum of the I-PSS questionnaire	20.0 ± 9.1	19.6 ± 8.9	23.8 ± 10.1	0.055
Quality of Life	4.1 ± 1.5	4.0 ± 1.5	4.7 ± 1.5	0.065

**Fig 1:**
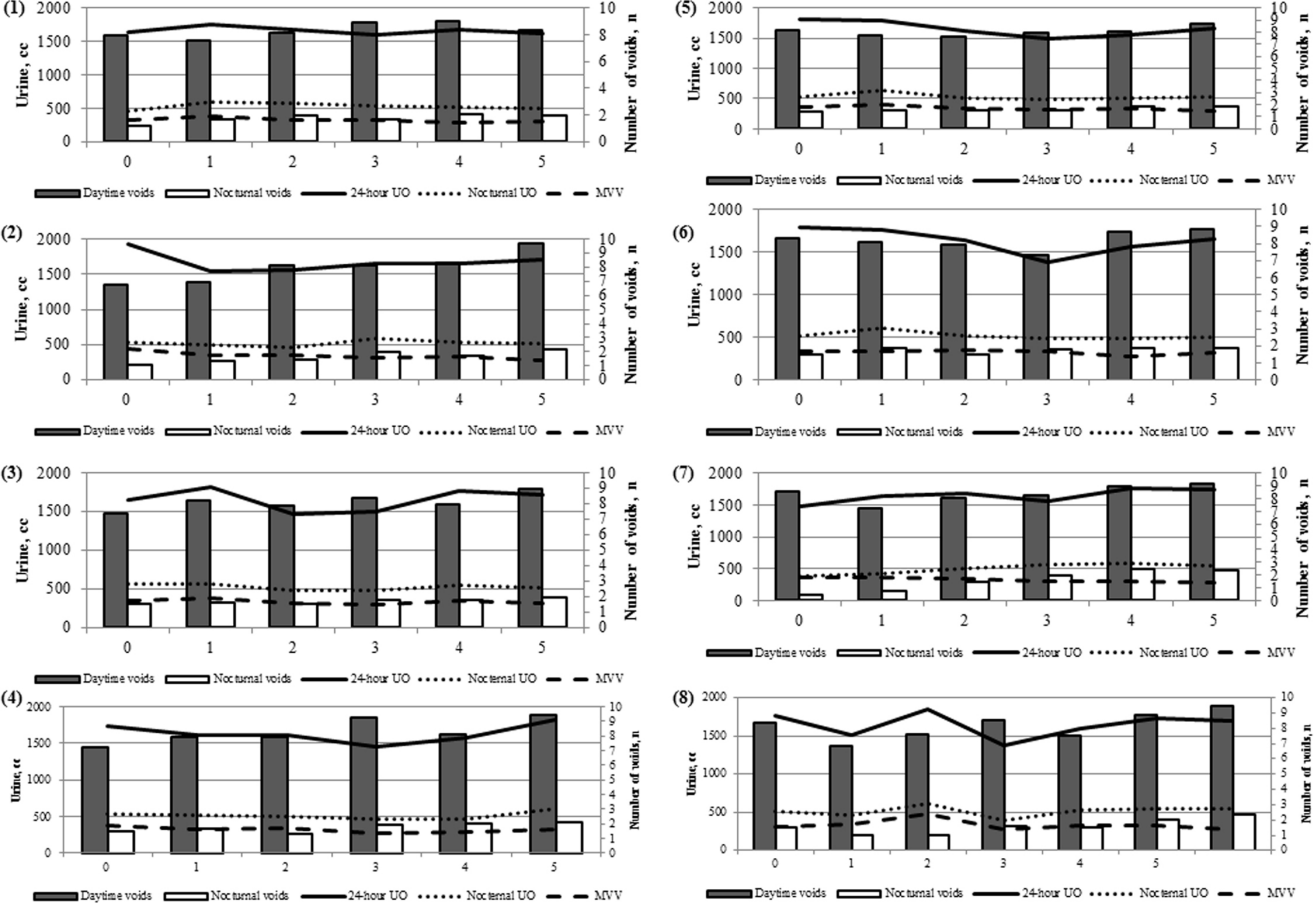
Relationship between each question in the I-PSS questionnaire and components of frequency volume chart. (1) Question 1 (24-hour UO; *p* = 0.950, Nocturnal UO; *p* = 0.379, MVV; *p* = 0.205, Daytime voids; *p* = 0.475, Nocturnal voids; *p* = 0.080). (2) Question 2 (24-hour UO; *p* = 0.537, Nocturnal UO; *p* = 0.518, MVV; *p* = 0.001, Daytime voids; *p*<0.001, Nocturnal voids; *p* = 0.001). (3) Question 3 (24-hour UO; *p* = 0.210, Nocturnal UO; *p* = 0.644, MVV; *p* = 0.109, Daytime voids; *p* = 0.250, Nocturnal voids; *p* = 0.467). (4) Question 4 (24-hour UO; *p* = 0.227, Nocturnal UO; *p* = 0.282, MVV; *p* = 0.032, Daytime voids; *p* = 0.010, Nocturnal voids; *p* = 0.055). (5) Question 5(24-hour UO; *p* = 0.556, Nocturnal UO; *p* = 0.488, MVV; *p* = 0.141, Daytime voids; *p* = 0.423, Nocturnal voids; *p* = 0.323). (6) Question 6 (24-hour UO; *p* = 0.265, Nocturnal UO; *p* = 0.377, MVV; *p* = 0.471, Daytime voids; *p* = 0.417, Nocturnal voids; *p* = 0.418). (7) Question 7 (24-hour UO; *p* = 0.629, Nocturnal UO; *p* = 0.041, MVV; *p* = 0.132, Daytime voids; *p* = 0.133, Nocturnal voids; *p*<0.001). (8) Quality of life (24-hour UO; *p* = 0.374, Nocturnal UO; *p* = 0.390, MVV; *p* = 0.006, Daytime voids; *p* = 0.021, Nocturnal voids; *p* = 0.003).

The MVV and the number of daytime voids were significantly related to Q2 (*p* = 0.001, *p* < 0.001), Q4 (*p* = 0.032, *p* = 0.010), and QoL (*p* = 0.006, *p* = 0.021) in the I-PSS, respectively (Fig 1). The number of nocturnal voids was associated with Q2 (*p* = 0.001), Q7 (*p* < 0.001), and QoL (*p* = 0.003) in the I-PSS. Nocturnal UO and NPi were related to Q7 (*p* = 0.041, *p* = 0.002) in the I-PSS (S1 Table). Ni was significantly associated with Q2 (*p* < 0.001) and Q7 (*p* < 0.001). NBCi was related to Q7 (*p* < 0.001) and QoL (*p* = 0.010). However, 24-hour UO was not associated with any question in the I-PSS questionnaire. In addition, the UO per body weight per hour was not associated with any question in the I-PSS questionnaire (Table 2).

**Table 2:** The relationship between each question in the I-PSS questionnaire with 24-hr Urine output / body weight.

	Score							
The I-PSS	0	1	2	3	4	5	6	*p*
Question 1	24.3 ± 10.9	26.8 ± 9.1	26.3 ± 9.3	23.8 ± 8.2	26.0 ± 12.4	25.0 ± 10.5	-	0.874
Question 2	29.2 ± 13.2	23.6 ± 9.0	24.9 ± 8.7	24.6 ± 7.8	24.6 ± 9.1	26.6 ± 12.5	-	0.571
Question 3	24.5 ± 9.5	27.0 ± 8.4	22.7 ± 7.6	22.8 ± 10.3	27.6 ± 10.2	27.0 ± 11.4	-	0.199
Question 4	26.2 ± 10.7	24.9 ± 8.7	25.8 ± 9.8	21.6 ± 6.3	23.4 ± 10.0	28.6 ± 12.7	-	0.114
Question 5	26.4 ± 10.4	27.7 ± 9.2	23.4 ± 8.3	23.3 ± 10.3	25.0 ± 9.9	25.7 ± 10.7	-	0.720
Question 6	26.9 ± 10.9	26.1 ± 9.4	24.9 ± 8.1	21.2 ± 5.7	24.6 ± 13.8	26.0 ± 10.1	-	0.494
Question 7	23.3 ± 11.4	23.6 ± 6.4	25.8 ± 10.8	24.2 ± 8.8	27.0 ± 12.1	27.7 ± 11.2	-	0.552
Quality of life	26.9 ± 14.2	22.6 ± 6.4	29.3 ± 9.7	21.2 ± 7.8	24.6 ± 9.0	26.0 ± 11.0	27.7 ± 11.5	0.315

Global polyuria was present in 19 patients (9.8%) (Table 1). No differences were found in patient characteristics, including age, body mass index, past medical history, prostate-specific antigen level, and prostate volume. The score for each question in the I-PSS questionnaire was higher in patients with global polyuria, although statistical significance was not reached, except for Q3 in I-PSS (2.8 vs. 3.7, *p* = 0.031). The number of daytime voids (8.0 vs. 11.2, *p* < 0.001) and the number of nocturnal voids (1.7 vs. 2.5, *p* = 0.002) were significantly higher in patients with global polyuria despite the large MVV (315.4 vs. 399.5 mL, *p* = 0.009).

**Fig 2:**
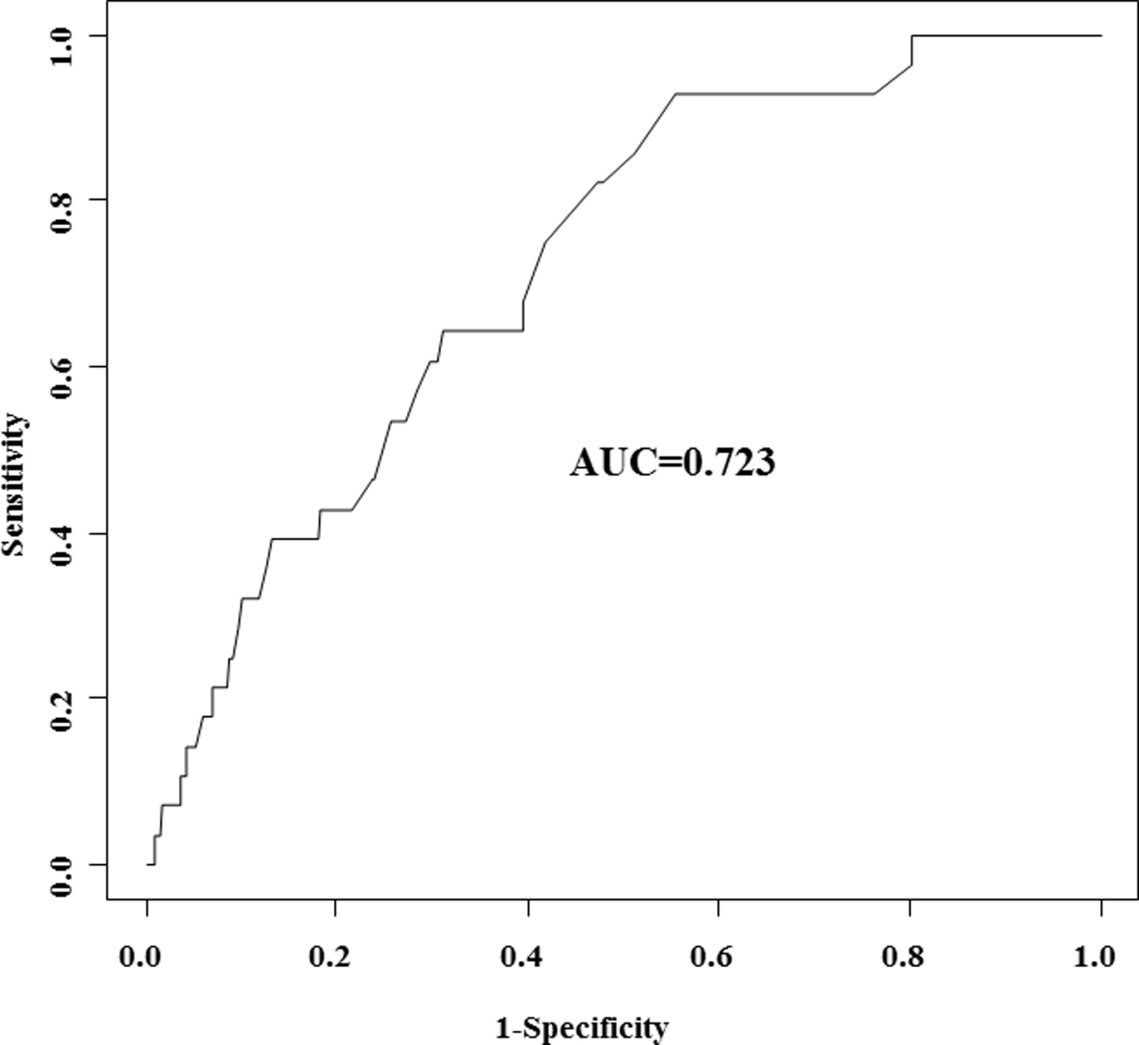
ROC curves of the prediction model for the presence of global polyuria.

In univariate analysis, Q3 in the I-PSS questionnaire, the number of total voids, the number of daytime voids, and the number of nocturnal voids were significantly related to the presence of global polyuria (Table 3). In multivariable analysis, the presence of diabetes mellitus [odds ratio (OR): 3.497, *p* = 0.039] and increased number of total voids (OR: 1.320, *p* < 0.001) were determined as significant predictors of global polyuria in BPH patients with LUTS. External validation of the model was performed using 397 patients with BPH, and the ROC curves of the prediction model for the presence of global polyuria are shown in Fig 2. The AUC of the prediction model was 0.723.

**Table 3:** Variables predicting global polyuria: Univariate and multivariable analysis.

	Univariate		Multivariable	
	OR (95% CI)	*p*	OR (95% CI)	*p*
Age	1.044 (0.976–1.117)	0.209		
Body mass index	0.862 (0.737–1.008)	0.063		
Diabetes (yes vs. no)	2.215 (0.780–1.008)	0.063	3.497 (1.067–11.46)	0.039
Hypertension (yes vs. no)	0.494 (0.170–1.433)	0.194		
Prostate volume	1.005 (0.989–1.021)	0.528		
The I-PSS questionnaire				
Question 1	1.081 (0.828–1.411)	0.569		
Question 2	1.330 (0.964–1.835)	0.083		
Question 3	1.382 (1.019–1.874)	0.037		
Question 4	1.273 (0.966–1.678)	0.086		
Question 5	1.284 (0.916–1.802)	0.147		
Question 6	1.079 (0.837–1.390)	0.559		
Question 7	1.427 (0.988–2.060)	0.058		
Sum of I-PSS	1.056 (0.988–1.119)	0.060		
QoL	1.497 (0.967–2.316)	0.070		
Number of voids				
Total	1.292 (1.143–1.461)	<0.001	1.320 (1.159–1.503)	<0.001
Daytime	1.336 (1.153–1.547)	<0.001		
Nocturnal	1.775 (1.217–2.588)	0.003		

## Discussion

The I-PSS questionnaire has been considered the baseline tool for assessing the subjective severity of LUTS in male patients owing to its ease of use and reliability [10]. In contrast to using the I-PSS questionnaire, FVC assessment cannot be easily applied in every patient because it needs to be performed for at least a few days to achieve reliable results [15, 16]. Moreover, as only a few studies have investigated the characteristics of patients who can benefit from FVC assessment in addition to I-PSS assessment, the use of the FVC has been determined by clinicians without objective criteria. In the current study, we assessed the information that could be obtained from the FVC and could not be inferred from the results of the I-PSS questionnaire in order to evaluate the usefulness of the FVC over the I-PSS questionnaire for patients with BPH. Moreover, we assessed the characteristics of patients who could benefit from undergoing FVC assessment in addition to I-PSS assessment, besides patients with a predominant storage symptom or nocturia.

This study showed that the results of the I-PSS questionnaire were generally reliable and correlated with the information obtained from the FVC. In this regard, the results of the FVC could be largely predicted using the results of the I-PSS questionnaire. In other words, the results of the current study can be useful for patients with BPH who cannot undergo FVC assessment owing to personal reasons and/or medical conditions. However, the results of the I-PSS should be interpreted with caution because they did not exactly match the results of the FVC. For example, the I-PSS slightly overestimated the number of nocturnal voids in accordance with the results of previous studies [17, 18], and the number of daytime voids was also slightly overestimated.

According to the results of this study, 24-hour UO and hourly UO/body weight cannot be inferred using the results of the I-PSS questionnaire. In other words, it is impossible to selectively perform FVC assessment based on the results of the I-PSS questionnaire, in order to identify patients with increased UO. In addition, the presence of global polyuria cannot be adequately predicted based on the I-PSS questionnaire (S1 Fig). Therefore, the presence of global polyuria should be suspected by clinicians on the basis of the patients’ symptoms and clinical characteristics, although FVC assessment needs to be performed to confirm the presence of global polyuria. The results of the current study suggest that global polyuria is present in approximately 10% of patients with BPH. Moreover, these patients generally presented with more severe LUTS than those without global polyuria. However, most clinicians are more interested in objective examinations such as prostate volume or uroflowmetry than in the fluid intake of their patients, although many patients have a large intake of fluids because of erroneous health beliefs or other reasons. Therefore, clinicians should take greater interest in their patients’ fluid intake to properly assess the cause of LUTS in patients with BPH. Moreover, it might be helpful in reducing the probability of overtreatment.

The characteristics of patients with global polyuria have not been well evaluated until now. According to the results of this study, diabetes mellitus and total number of voids were significant predictors of global polyuria in patients with BPH and LUTS. In addition, these results could be reliable because the prediction model for global polyuria was externally validated and showed an AUC of >0.7. In a previous study, diabetes mellitus was reported to be strongly related to LUTS [19]. The results of the current study suggest that the relationship between diabetes mellitus and LUTS might come from the increased UO in patients with diabetes, in addition to other diabetes-related neurologic changes. Moreover, 2 recent studies reported that bladder function could be affected by polyuria itself in diabetic patients [20, 21]. In this regard, UO should be carefully monitored in diabetic patients and the FVC could be a useful tool for monitoring UO in order to protect the normal bladder function. Although the present study could not assess the reason for the increased proportion of global polyuria in patients with diabetes, uncontrolled diabetes, changes in lower urinary tract function, and increased fluid intake due to concerns about renal function could be the possible reasons for the increased UO in diabetic patients with BPH. Therefore, it is necessary to educate BPH patients with diabetes about adequate water intake.

In this study, the number of total voids was also determined as a predictor of global polyuria. As mentioned above, current clinical guidelines only recommend FVC assessment in patients with a predominant storage symptom or nocturia [10]. Storage symptom and nocturia (Q2, Q4, and Q7 in the I-PSS questionnaire) were significantly associated with the information obtained from the FVC, which supports the current clinical guidelines. However, according to the current study results, patients with increased number of voids, regardless of storage symptom, can be potential candidates for FVC assessment to detect the presence of global polyuria. Because patients with global polyuria showed increased MVV [22], storage symptom would not be present in these patients. In other words, FVC assessment needs to be considered in BPH patients with diabetes mellitus and increased number of voids to accurately detect the presence of global polyuria although storage symptom was not predominant. In addition, the FVC was reported to be useful for the improvement of LUTS if appropriate lifestyle modification can be applied [23, 24]. To summarize, the FVC could be useful not only for identifying patients with global polyuria but also for improving LUTS. Therefore, careful history taking about voiding symptoms should be performed and the number of total voids also needs to be assessed at the first visit to select appropriate candidates for FVC assessment, especially in patients with concomitant diabetes mellitus.

This study has several limitations, including its retrospective design and the small number of patients. However, the results of the current study are thought to be reliable because external validation was performed. The duration between FVC and I-PSS assessments could be another limitation of the current study although more than three-quarters of patients underwent FVC and I-PSS assessments within a month. In addition, the results of the current study need to be verified in Western countries before being widely applied [25]. Nevertheless, this study is valuable because it presents several novel findings, including the actual incidence of global polyuria and the patient characteristics predicting the presence of global polyuria, which can be helpful for clinicians in managing male patients with LUTS.

## Conclusions

Global polyuria is present in about 10% of patients with BPH and LUTS, and it can worsen LUTS. However, global polyuria cannot be predicted using the results of the I-PSS questionnaire. In this regard, the FVC needs to be used to accurately diagnose global polyuria in patients with BPH and LUTS. In the current study, diabetes mellitus and increased number of voids, which could be easily assessed through history taking, were determined as the predictors of global polyuria. Therefore, in addition to patients with a predominant storage symptom or nocturia, FVC assessment needs to be considered in diabetic patients with BPH and increased number of voids to rule out the presence of global polyuria.

## Supporting information

S1 Table The relationship between each question in I-PSS questionnaire and other obtainable information using frequency volume chart. (DOCX)

S1 Fig ROC curve for predicting global polyuria using each score of I-PSS questionnaire. (DOCX)
