# Supplementary material for: Usefulness of the frequency-volume chart over the International Prostate Symptom Score in patients with benign prostatic hyperplasia in view of global polyuria
Source: PLoS One. 2018 Jul 11;13(7):e0197818. doi: 10.1371/journal.pone.0197818 (PMC6040686; doi:10.1371/journal.pone.0197818)
Supplement: S1 Table — (DOCX) [file pone.0197818.s001.docx]

Supporting Table S1. The relationship between each question in I-PSS questionnaire and other obtainable information using frequency volume chart

1. NPi

Score

The I-PSS 0 1 2 3 4 5 6 *p*

Question 1 28.6 ± 13.1 33.1 ± 11.3 33.7 ± 10.2 32.8 ± 11.6 31.7 ± 10.3 31.1 ± 9.8 - 0.570

Question 2 26.7 ± 11.7 33.2 ± 12.9 29.0 ± 11.2 34.9 ± 10.1 31.7 ± 8.7 31.9 ± 9.2 - 0.107

Question 3 33.1 ± 12.8 30.4 ± 9.9 30.9 ± 12.6 32.1 ± 9.9 32.7 ± 10.3 30.8 ± 10.4 - 0.902

Question 4 31.3 ± 14.2 32.5 ± 10.1 31.1 ± 8.6 31.9 ± 11.1 28.8 ± 10.0 33.0 ± 10.6 - 0.735

Question 5 28.2 ± 11.9 33.2 ± 12.1 32.1 ± 11.3 32.4 ± 10.6 32.2 ± 13.1 31.5 ± 10.0 - 0.829

Question 6 28.5 ± 11.1 34.8 ± 12.1 32.4 ± 9.5 34.8 ± 12.7 31.6 ± 9.8 30.3 ± 10.0 - 0.143

Question 7 24.6 ± 6.1 26.1 ± 9.8 31.8 ± 10.6 34.8 ± 9.8 34.1 ± 11.0 29.8 ± 12.7 - 0.002

Quality of life 25.7 ± 13.0 32.1 ± 1.8 32.3 ± 7.0 29.6 ± 9.0 33.1 ± 1.6 30.9 ± 9.6 32.7±13.7 0.645

1. Ni

Score

The I-PSS 0 1 2 3 4 5 6 *p*

Question 1 1.40 ± 0.60 1.62 ± 0.61 1.77 ± 0.76 1.79 ± 0.82 0.97 ± 1.00 1.69 ± 0.66 - 0.118

Question 2 1.26 ± 0.70 1.47 ± 0.61 1.41 ± 0.56 1.96 ± 0.72 1.69 ± 0.80 1.95 ± 0.81 - <0.001

Question 3 1.67 ± 0.88 1.60 ± 0.48 1.57 ± 0.83 1.79 ± 0.90 1.74 ± 0.74 1.73 ± 0.66 - 0.876

Question 4 1.45 ± 0.71 1.75 ± 0.80 1.52 ± 0.60 1.83 ± 0.79 1.64 ± 0.61 1.94 ± 0.82 - 0.073

Question 5 1.53 ± 0.80 1.72 ± 0.67 1.57 ± 0.77 1.63 ± 0.68 1.69 ± 0.78 1.77 ± 0.77 - 0.774

Question 6 1.64 ± 0.81 1.88 ± 0.80 1.66 ± 0.71 1.54 ± 0.51 1.85 ± 1.04 1.65 ± 0.59 0.519

Question 7 1.04 ± 0.44 1.21 ± 0.50 1.61 ± 0.60 1.95 ± 0.83 1.93 ± 0.75 0.89 ± 0.78 <0.001

Quality of life 1.64 ± 1.10 1.36 ± 0.50 1.35 ± 0.43 1.57 ± 0.64 1.66 ± 0.67 0.79 ± 0.82 1.96±0.83 0.202

1. NBCi

Score

The I-PSS 0 1 2 3 4 5 6 *p*

Question 1 0.78 ± 0.65 1.03 ± 0.56 1.05 ± 0.52 0.87 ± 0.81 1.07 ± 0.85 1.21 ± 0.78 - 0.164

Question 2 0.87 ± 0.56 0.86 ± 0.68 0.96 ± 0.65 1.00 ± 0.53 1.04 ± 0.71 1.25 ± 0.89 - 0.175

Question 3 0.84 ± 0.54 0.99 ± 0.63 0.90 ± 0.65 1.01 ± 0.74 1.11 ± 0.96 1.18 ±0.74 - 0.347

Question 4 1.01 ± 0.48 0.96 ± 0.61 0.80 ± 0.62 1.07 ± 0.88 1.31 ± 0.93 1.14 ± 0.74 - 0.150

Question 5 0.90 ± 0.44 0.80 ± 0.71 0.91 ± 0.59 0.88 ± 0.67 1.18 ± 0.97 1.12 ± 0.73 - 0.299

Question 6 0.84 ± 0.45 0.94 ± 0.70 0.85 ± 0.48 1.22 ± 0.46 1.09 ± 1.08 1.23 ± 0.79 - 0.061

Question 7 0.44 ± 0.33 0.60 ± 0.44 0.87 ± b0.51 1.04 ± 0.56 1.51 ± 1.00 1.50 ± 0.71 - <0.001

Quality of life 0.86 ± 0.35 0.64 ± 0.51 0.69 ± 0.55 1.07 ± 0.75 0.88 ± 0.58 1.18 ± 0.81 1.40±0.76 0.010
