# Supplementary material for: Usefulness of the frequency-volume chart over the International Prostate Symptom Score in patients with benign prostatic hyperplasia in view of global polyuria
Source: PLoS One. 2018 Jul 11;13(7):e0197818. doi: 10.1371/journal.pone.0197818 (PMC6040686; doi:10.1371/journal.pone.0197818)

Supporting Figure S1. ROC curve for predicting global polyuria using each score of I-PSS questionnaire


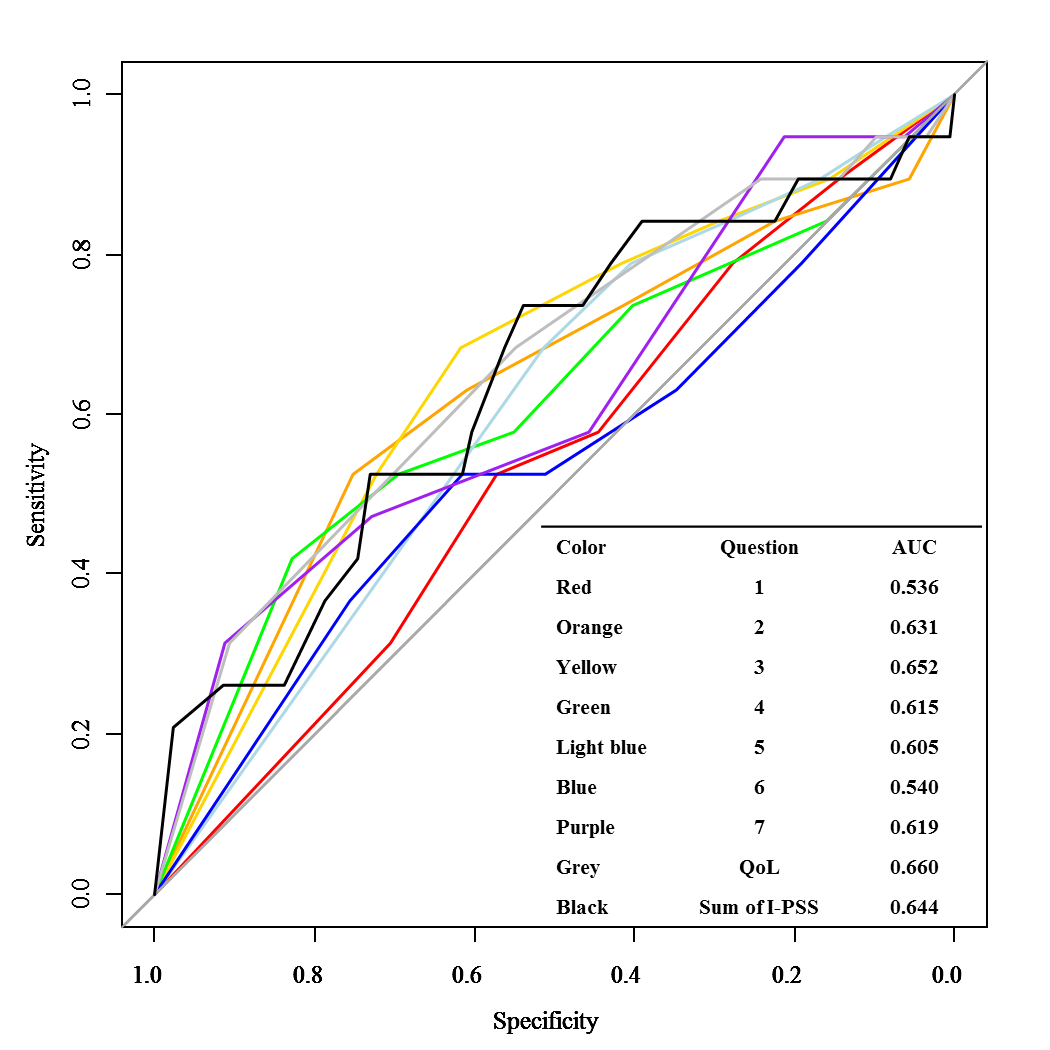

Supplement: S1 Fig — (DOCX) [file pone.0197818.s002.docx]
